# Growth and Assemblage Dynamics of Temperate Forest Tree Species Match Physiological Resilience to Changes in Atmospheric Chemistry

**DOI:** 10.1111/gcb.70147

**Published:** 2025-03-26

**Authors:** Filip Oulehle, Pavel Šamonil, Otmar Urban, Josef Čáslavský, Alexander Ač, Ivana Vašíčková, Jakub Kašpar, Pavel Hubený, Rudolf Brázdil, Miroslav Trnka

**Affiliations:** ^1^ Czech Geological Survey Prague Czech Republic; ^2^ Global Change Research Institute of the Czech Academy of Sciences Brno Czech Republic; ^3^ Department of Forest Ecology The Silva Tarouca Research Institute Brno Czech Republic; ^4^ Faculty of Forestry and Wood Technology Mendel University in Brno Brno Czech Republic; ^5^ Šumava National Park Vimperk Czech Republic; ^6^ Institute of Geography Masaryk University Brno Czech Republic

**Keywords:** CO_2_ fertilization, nitrogen deposition, stable isotopes, tree rings, water‐use efficiency

## Abstract

Human‐induced environmental changes are altering forest productivity and species composition, significantly impacting tree physiology, growth, water uptake, and nutrient acquisition. Investigating the intricate interplay between plant physiology and environmental shifts, we analyzed tree‐ring isotopes (δ^13^C, δ^18^O, and δ^15^N) to track long‐term trends in intrinsic water‐use efficiency (iWUE) and nitrogen availability for European beech, Norway spruce, and silver fir in a unique old‐growth temperate mountain forest since 1501 ce. Our findings reveal that Norway spruce, a dominant species, exhibited iWUE saturation, exacerbated by acidic precipitation, resulting in growth declines during periods of high acidic air pollution and increased drought frequency. In contrast, deep‐rooted, deciduous European beech demonstrated physiological resilience to acid deposition, benefiting from lower dry deposition of precipitation acidity and thriving under conditions of increased nitrogen deposition and elevated air temperatures, thereby sustaining stem growth regardless of potential climatic limitations. Silver fir showed the most dynamic response to acidic air pollution, with contemporary adaptations in leaf gas exchange allowing accelerated stem growth under cleaner air conditions. These different species responses underscore shifts in species competition, with European beech gaining dominance as Norway spruce and silver fir decline. Furthermore, the influence of ontogeny is evident, as tree‐rings exhibited lower initial iWUE values and higher δ^15^N, reflecting changes in nitrogen uptake dynamics and the ecological role of tree age. Our study integrates tree‐growth dynamics with physiological and nutrient availability trends, revealing the pivotal role of atmospheric chemistry changes in shaping the competitive dynamics and long‐term growth trajectories of dominant tree species in temperate forests.

## Graphical abstract

This study examines how environmental changes over centuries have impacted trees in natural mountain forest. Stable isotopes in tree rings reveal shifts in carbon and water exchanges (iWUE) and nitrogen cycling (δ15N). Tree species react differently to pollution and climate change: European beech benefits from more nitrogen and warming, while Norway spruce suffers from iWUE saturation. Silver fir's physiological functioning has improved with better air quality. Understanding these species‐specific responses is crucial for predicting how forests will respond to ongoing environmental changes, assessing their vulnerability, and determining their future potential for carbon storage.

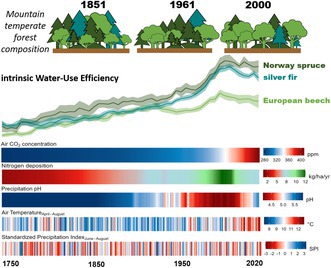

## 1 Introduction

Tree growth in moist forests has accelerated over the last century (McMahon et al. 2010a; Fang et al. 2014; Pretzsch et al. 2014, 2023; Hogan et al. 2024). The primary drivers behind this trend remain uncertain, with possibilities including the rising concentration of atmospheric CO_2_, which enhances photosynthesis, climate change that causes warming and prolongs the growing season (although a longer growing season does not necessarily entail higher growth as it could lead to drier conditions), and chronic nitrogen (N) deposition contributing to eutrophication (Laubhann et al. 2009; McMahon et al. 2010a; Schimel et al. 2015; Etzold et al. 2020; Davis et al. 2022; Clark et al. 2023). Additionally, it is unclear if increased forest growth will sustain a long‐term carbon (C) sink, if biomass accumulation will reach stoichiometric limits, or if ecosystem structure and function will be altered due to changes in the frequency and magnitude of disturbances (Körner 2015; Senf et al. 2018; Brienen et al. 2020; Brodribb et al. 2020; McDowell et al. 2020; Duffy et al. 2021; Sharma et al. 2023).

During the Holocene, the moist temperate mixed forest of central Europe was dominated by Norway spruce ( *Picea abies* (L.) Karst.), joined by European beech ( *Fagus sylvatica* L.) and later by silver fir ( *Abies alba* L.) (Carter et al. 2018). A gradual increase in beech proportion altered the disturbance regime from fire to wind and biotic disturbances around 6000 years BP (Bobek et al. 2019). Trees significantly influenced hillslope processes through intense uprooting dynamics, to which the dominant spruce is particularly sensitive (Šamonil et al. 2023). In the last two centuries, natural forests have mostly been converted into managed ones featuring homogeneous structure and species composition (Mottl et al. 2021). In many parts of Europe, these managed forests are often dominated by Norway spruce (Johann et al. 2004). This homogenization increases vulnerability to climate change consequences such as high temperatures, droughts, fires, and biotic disturbances (Seidl et al. 2011; Forzieri et al. 2021; Hlásny et al. 2021; Patacca et al. 2023). The scarcity of structurally and species‐diverse old‐growth temperate forests in Europe makes them a valuable yet underrepresented source of information for understanding forest responses to environmental changes.

**FIGURE 1:**
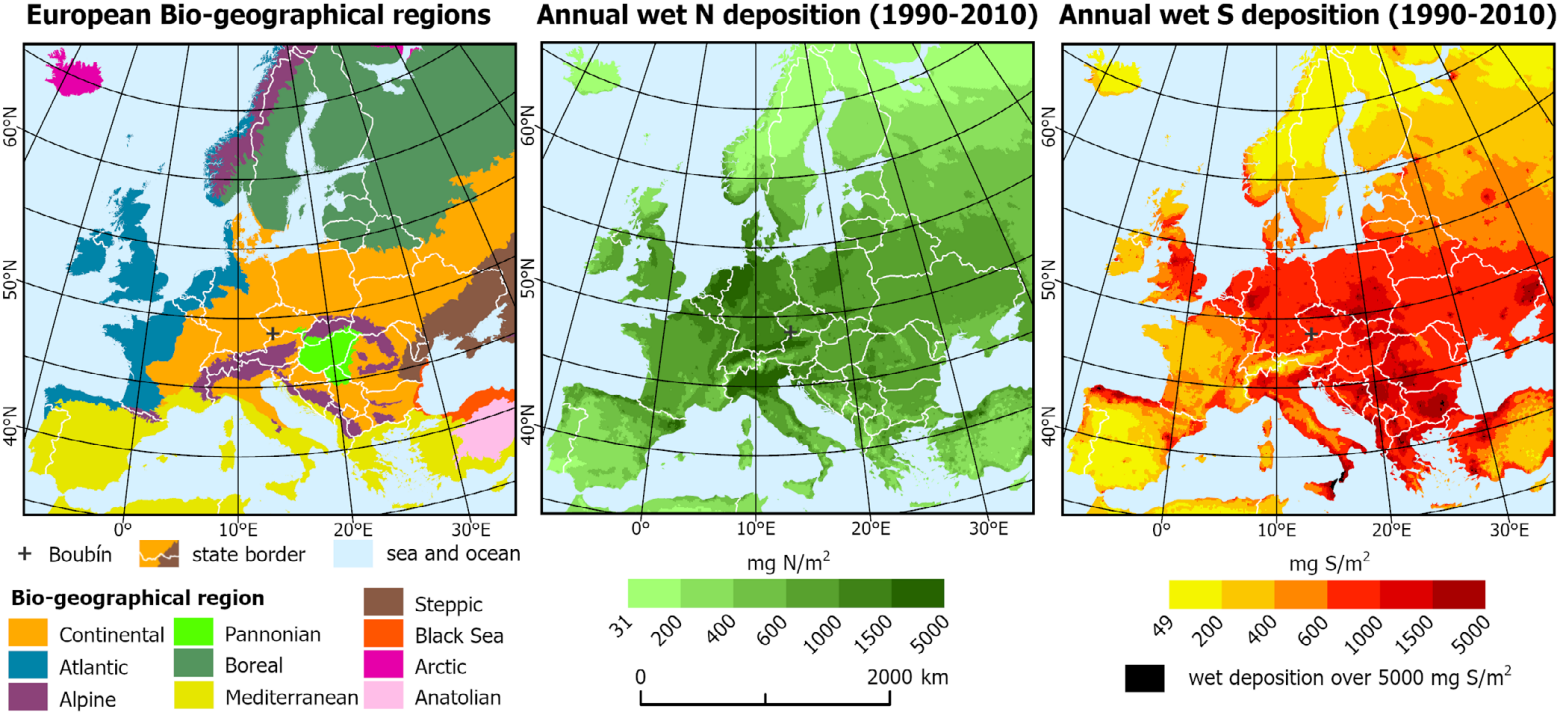
Geographical distribution of European biogeographical regions (EEA 2017) and representation of average nitrogen (N) and sulphur (S) deposition on the European continent. The study site is marked with a black cross. Wet deposition data represent modelled average annual deposition between 1990 and 2010 in mg N(S) m^−2^ year^−1^ (EMEP 2023). Map lines delineate study areas and do not necessarily depict accepted national boundaries.

This study focuses on the Boubín Primeval Forest Reserve, a historically uncut, mixed montane primeval forest protected since 1858, serving as a benchmark for continental European mountain forests (Sabatini et al. 2021). While being impacted by air pollution (EMEP 2023) (Figure 1), the reserve boasts unique forest inventory records dating back to 1851 (Šebková et al. 2011), revealing a rising dominance of European beech at the expense of Norway spruce, alongside a significant decline in silver fir during the 20th century. These structural changes reflect broader forest dynamics (Ma et al. 2023) and a successional shift from shallow‐rooted conifers to deep‐rooted deciduous trees. This shift is likely influenced by several interacting factors, including soil hydromorphism gradients, where Norway spruce thrives in wetter soils and exhibits lower drought tolerance, while European beech is more adapted to well‐drained soils (Daněk et al. 2019). Additionally, the long‐term effects of atmospheric pollution may alter soil nutrient availability through soil acidification and N enrichment (Oulehle et al. 2024).

Tree growth depends on the physiological state of the plant and nutrient availability (Körner 2015; Delpierre et al. 2016; Cabon et al. 2022). Recent findings indicate that during experimental drought events, European beech acclimates better and recovers faster than spruce, particularly benefiting smaller trees in growth (Motte et al. 2023). While spruce adapts by significantly reducing water use and leaf area, beech maintains its leaf area and reduces water usage, enhancing its drought recovery (Hesse et al. 2024). Moreover, mixed stands of fir and beech exhibit greater resistance and productivity under extreme drought than monospecific stands, highlighting their potential for sustainable forest management amid climate change (Gbur et al. 2025).

As trees age, they exhibit increased secondary growth, leading to the development of larger stems, branches, and extensive root systems. This enhanced architecture significantly influences both water use and N uptake. For instance, older trees often possess more extensive root networks that access deeper soil layers, improving water uptake during dry events and enhancing drought resilience (Sperry et al. 2002; Claus and George 2011). These mature root systems can also enhance the uptake of nitrogen by accessing lower nutrient horizons and maximizing contact with mycorrhizal fungi, which are critical for nutrient acquisition. Consequently, the allocation of resources to secondary growth can improve the overall hydraulic efficiency of older trees, allowing them to optimize water transport from roots to leaves, ultimately influencing stomatal performance and transpiration rates (Ryan et al. 2000).

In this context, analyzing isotopic ratios in tree rings provides critical insights into past physiological responses to environmental changes. Using ^13^C/^12^C and ^15^N/^14^N isotope ratios in the tree rings of European beech, Norway spruce, and silver fir, we reconstructed intrinsic water‐use efficiency (iWUE_wood_) and N availability. The iWUE reflects the trade‐off between photosynthetic CO_2_ uptake (*A*_net_) and stomatal conductance to water (g_s_) (Farquhar et al. 1989; Saurer, Cherubini, et al. 2004; Lavergne et al. 2019; Mathias and Thomas 2021). Trends in N availability were archived in tree rings as a δ^15^N proxy (Kranabetter et al. 2021; Oulehle et al. 2022; Savard et al. 2023), with tree‐ring δ^15^N reflecting trends in stand N limitation (McLauchlan and Craine 2012; Craine et al. 2018). An increasing tree‐ring δ^15^N signature indicates an opening of the N cycle, influenced by disturbance or chronic N deposition (Saurer, Siegwolf, et al. 2004; Savard 2010; Guerrieri et al. 2011; Kranabetter et al. 2021). The three species analyzed—European beech, Norway spruce, and silver fir—are all ectomycorrhizal types that significantly influence their nitrogen uptake and δ^15^N signals (Hobbie et al. 1999). Their specific interactions with mycorrhizal fungi lead to variations in nitrogen assimilation, reflected in differing δ^15^N values that indicate varying levels of nitrogen availability (Högberg et al. 2011). Moreover, site‐specific soil characteristics, such as texture and bedrock composition, can impact moisture and pH levels, adding complexity to the interpretation of δ^15^N signals (Savard et al. 2023).

This δ^13^C and δ^15^N combined isotope chronology was enhanced by analyzing δ^13^C and δ^18^O (^18^O/^16^O) isotope ratios in α‐cellulose from annually resolved spruce tree rings. While we focused on bulk wood for nitrogen analysis, since nitrogen is not found in cellulose and its low concentration in wood necessitated combining data from several tree rings for a thorough assessment, we measured oxygen isotopes in cellulose for each individual tree ring of spruce, alongside carbon isotopes. The combination of calculated iWUE and δ^18^O from cellulose permits an independent qualitative evaluation of the contributions of *A*_net_ and g_s_ to changes in iWUE. This is because the calculated ^18^O enrichment in leaf water above the source water (Δ^18^O_lw_) reflects transpiration and g_s_ variability (Guerrieri et al. 2019; Mathias and Thomas 2021; but see Lin et al. 2022). Our dual measurement approach, exclusive to spruce as the dominant species in the studied ecosystem, enables a more refined understanding of spruce physiological responses to environmental changes. Long‐term trends in forest growth and water use efficiency are shaped by the complex interplay of species‐specific physiological traits and environmental factors, with nitrogen deposition emerging as a key driver (Adams et al. 2021). This long‐term nitrogen fertilization effect differentially impacts species due to their distinct physiological strategies and nitrogen uptake mechanisms. Beech, with efficient nutrient acquisition, benefits from increased nitrogen and CO_2_, resulting in enhanced growth and resilience (Gharun et al. 2021). However, this response is modulated by water availability and the complex interaction between net photosynthesis and stomatal conductance, and leaf N content significantly affects this coordination (Aranda et al. 2017). Conifers, conversely, exhibit stomatal adjustments to elevated CO_2_, further impacted by acid deposition, leading to reduced growth (Šantrůčková et al. 2007). In addition, inherent differences in leaf architecture and stomatal control mechanisms (isohydric vs. anisohydric responses) between deciduous beech and both conifers contribute to their differing iWUE responses (Mathias and Thomas 2021).

Centennial trends in iWUE, δ^15^N, and basal area increment in major tree species are influenced by the combined effects of species‐specific physiological responses to changing environmental conditions, especially variations in atmospheric CO_2_ and nitrogen availability. Interactions between physiological traits and environmental factors shape these long‐term trends. We hypothesize that higher growth dynamics in beech are driven by its ability to exploit the long‐term fertilizing effects of nitrogen and CO_2_. In contrast, conifers' adjustment of stomatal conductance to increasing CO_2_, exacerbated by acid deposition, primarily causes growth decline and increased sensitivity to drought.

## 2 Materials and Methods

### 2.1 Study Site and Tree Sampling

The Šumava Mts., also known as the Bavarian Forest, straddles the border between the Czech Republic, Germany, and Austria and covers an area of 1671 km^2^, making it the largest forested mountain range in Central and Western Europe. Consisting mainly of acidic rocks, with the highest peak being Großer Arber at 1456 m asl, the range has average annual temperatures of around 5°C at 750 m, dropping to around 3°C at 1200 m. Annual precipitation is variable, with the peaks and main ridges receiving around 1600 mm, while the rain shadow areas on the north‐eastern slopes receive an average of 800–900 mm.

Two national parks have been established to protect the pristine mountain spruce and mixed spruce‐beech forests in the area. The trees analyzed in this study originated from these old‐growth forests. Dendrochronological analysis revealed a complex disturbance regime (Kašpar et al. 2021), including fine‐scale gap dynamics and occasional severe disturbances, such as windstorms and bark beetle outbreaks. Historically, fire events were dominant (Bobek et al. 2019), but severe disturbances have increased in recent decades, reflecting trends observed across Europe (Seidl et al. 2014).

Since 2011, the research team at the Department of Forest Ecology (DFE) has extracted and analyzed more than 7000 core series from trees in the natural and managed forest ecosystems of the Šumava Mts. (Kašpar et al. 2021; Vašíčková et al. 2021) These cores were obtained from both living trees and freshly uprooted or broken trees due to windstorms, biotic attacks (e.g., bark beetles or fungi), and other disturbances. For this study, we selected core samples from an extensive collection representing European beech, Norway spruce, and silver fir. For each species, we randomly selected 8–12 of the oldest individuals growing in mixed stands. One to four cores were extracted from each selected tree at a height of 0.5–1.0 m above ground level. Such ancient trees are rare, constituting only about 1% of even natural forests, and are completely absent from managed forests. Where possible, we re‐sampled these individuals using a wider 12 mm diameter Haglof increment borer.

All sampled trees were located within the Šumava region at altitudes of 800–1200 m asl, ensuring consistent climatic conditions across all sampling sites. Our sample collection included trees from the Boubín Primeval Forest Reserve (48.97778°N, 13.81073°E, 666 ha), Jilmová skála Forest Reserve (48.95356°N, 13.79752°E), Zátoňská Hora Forest Reserve (48.94426°N, 13.82988°E), and Otov Forest Reserve (48.63537°N, 14.05890°E). Additionally, we included very old cuttings from other researchers and foresters collected over the past 20 years. All sampled trees were exposed to sunlight at the time of sampling.

### 2.2 Dendrochronological Analyses

In the dendrochronological laboratory of the DFE, the samples were ground with 400‐grit sandpaper and then scanned with an EPSON LA 2400 scanner at a resolution of 1600 DPI. The WinDENDRO 2022 software was used to automatically determine the width of the annual rings to an accuracy of 0.01 mm. This was followed by cross‐dating, in which the measured tree‐ring series were compared with each other using PAST 5 (SCIEM, 2007) and COFECHA (Holmes 1983) software, and with a standard species‐specific chronology that represents the general average growth of tree species, eliminating age trend and local growth plasticity (SI Table 1). At this stage, the final correction of the measured data takes place, eliminating possible errors due to imprecise automatic detection and the occasional occurrence of missing or false tree rings. The disturbance history was analyzed in detail at the level of the individual tree. In other words, the moments when the growth space of the analyzed tree was released by the removal of the overstory tree were recorded. The sudden growth changes that exceeded 50% of the boundary line value (Black and Abrams 2004) were considered a release. If the tree was growing in the gap below the open canopy when it was young (so‐called gap origin), this was also recorded. Disturbance history was analyzed according to Šamonil et al. (2013).

**TABLE 1:** Parameter estimates (including interaction with air CO_2_) of the average linear mixed effects (LME) model identifying environmental factors influencing iWUE of European beech, Norway spruce and silver fir since 1501 ce. The LME model results shown are for the average conditional model, according to parameters for the final models of European beech (*N* = 16), Norway spruce (*N* = 11) and silver fir (*N* = 8) (models with ΔAIC_c_ < 2 derived from the best model with the lowest AIC_c_). *R* ^2^ values shown are for the best model; the marginal *R* ^2^ describes the variance in iWUE explained by fixed effects only, while the conditional *R* ^2^ represents the variance explained by fixed and random effects.

Predictors	European beech iWUE_wood_			Norway spruce iWUE			silver fir iWUE_wood_		
	Estimate ± SE	*z*‐value	*p*‐value	Estimate ± SE	*z*‐value	*p*‐value	Estimate ± SE	*z*‐value	*p*‐value
Intercept (μmol mol^−1^)	37.09 ± 2.36	15.7	< 0.001	50.18 ± 3.26	15.4	< 0.001	47.19 ± 1.83	25.8	< 0.001
α‐cellulose	—	—	—	9.55 ± 5.04	1.89	0.058	—	—	—
Cambial age (year)	8.3 ± 2.71	3.06	0.002	3.87 ± 1.36	2.85	0.004	6.44 ± 1.62	3.98	< 0.001
Air CO_2_ (ppm)	2.49 ± 1.96	1.27	0.205	8.87 ± 1.08	8.20	< 0.001	4.99 ± 1.08	4.62	< 0.001
N deposition (kg ha^−1^ year^−1^)	5.23 ± 1.87	2.80	0.005	5.06 ± 1.55	3.27	0.001	5.86 ± 1.19	4.91	< 0.001
pH precipitation	1.07 ± 1.03	1.04	0.299	1.42 ± 1.07	1.33	0.183	−1.40 ± 1.12	1.24	0.214
SPI_Jun‐Aug_	0.11 ± 0.11	0.94	0.345	0.24 ± 0.19	1.27	0.205	−0.14 ± 0.13	1.05	0.295
*T*_Apr‐Aug_ (°C)	−0.15 ± 0.19	0.78	0.434	—	—	—	−0.26 ± 0.21	1.22	0.224
Air CO_2_: Cambial age	−1.39 ± 1.27	1.10	0.273	−1.42 ± 0.54	2.64	0.008	−0.96 ± 0.82	1.16	0.244
Air CO_2_: N deposition	−2.07 ± 1.06	1.96	0.049	2.20 ± 0.92	2.39	0.017	−0.88 ± 1.09	0.81	0.417
Air CO_2_: pH precipitation	—	—	—	−1.98 ± 0.70	2.81	0.005	−1.21 ± 0.66	1.82	0.069
Air CO_2_: SPI_Jun‐Aug_	—	—	—	0.46 ± 0.24	1.93	0.054	—	—	—
Observations	832			948			677		
Marginal *R* ^2^	0.67			0.74			0.78		
Conditional *R* ^2^	0.79			0.90			0.85		

Abbreviations: *p*‐value, two‐tailed; SE, standard error; *z*‐value, Wald chi‐squared test.

### 2.3 Cellulose Extraction and Isotope Analysis in Spruce Tree Rings

We analyzed annual cellulose samples solely from Norway spruce, the dominant species in the forest composition. Absolutely dated and annually separated tree rings from five individual trees were cut into small pieces and placed in F57 Teflon bags (Ankom Technology, Macedon, NY, USA) for α‐cellulose extraction following Boettger et al. (2014). Each tree was sampled individually, without pooling across years or individuals. To ensure comparability, the sampled trees exhibited comparable dasometric characteristics (see Table S1).

For cellulose extraction, Teflon bags containing tree‐ring samples were washed twice in a 5% NaOH solution at 60°C for 2 h, followed by a further wash in a 7% NaClO_2_ solution at 60°C for a further 30 h. Acetic acid (99.8%) was added to the solution to maintain the pH at 4–5. The Teflon bags were then washed three times in hot distilled water (90°C). This method efficiently extracts α‐cellulose from uncontaminated or chemically treated wood cores (Urban et al. 2021). The extracted cellulose was dried at 50°C for 24 h, sealed in Eppendorf microtubes, and stored in the dark at 21°C until analysis.

To determine the stable isotope ratios of carbon (^13^C/^12^C) and oxygen (^18^O/^16^O) in tree‐ring cellulose, approximately 1.0 mg of homogenized α‐cellulose was weighed into silver capsules (Elementar Analysensysteme, Langenselbold, Germany) and pyrolyzed at 1450°C to carbon monoxide (CO) using the high‐temperature furnace of an elemental analyzer varioPYRO cube (Elementar Analysensysteme, Germany). The ^13^C/^12^C and ^18^O/^16^O ratios in CO were determined using an ISOPRIME100 continuous flow isotope ratio mass spectrometer (Isoprime, Manchester, UK) and calibrated against International Atomic Energy Agency (IAEA) certified reference materials: benzoic acids (IAEA‐601 and IAEA‐602) and cellulose (IAEA_CH3). Finally, the δ^13^C_cellulose_ and δ^18^O_cellulose_ values in ‰ were related to the Vienna Pee Dee Belemnite (VPDB) and Vienna Standard Mean Ocean Water (VSMOW), respectively. The measurement accuracy of δ^13^C and δ^18^O ratios, determined as the standard deviation (σ) of six replicate measurements on the same homogenized α‐cellulose sample, was better than 0.06‰ and 0.17‰ for δ^13^C and δ^18^O, respectively.

### 2.4 Determination of δ^13^C and δ^15^N in Bulk Wood of Beech, Spruce, and Fir

We measured δ^13^C in both cellulose and bulk wood to provide a comprehensive assessment of isotopic variation across different sample types. As described in the previous section, cellulose samples were exclusively taken from Norway spruce. Due to the low N content in tree rings, bulk wood was pooled into 5‐year segments to ensure a sufficient N amount for precise isotope analysis. Pooled 5‐year segments of bulk wood (counted from the most recent year in the dataset) were analyzed from 12 European beech and 12 silver fir trees, while bulk wood samples from six Norway spruce trees were examined. Bulk wood segments were chopped and finely homogenized using an MM200 mill (Retsch, Haan, Germany). Although pooling across years can potentially bias the inter‐annual isotopic signals, this approach was necessary given the extensive temporal span of our dataset (500 years) and the number of trees sampled (35). This strategy ensured sufficient material for meaningful isotopic analysis while maintaining the integrity with the spruce dataset described earlier.

For isotope analysis, samples were weighed into tin capsules (approximately 1.5 for δ^13^C and 20 mg for δ^15^N) and combusted at 960°C using a varioPYRO cube elemental analyzer (Elementar Analysensysteme, Germany). The ^13^C/^12^C and ^15^N/^14^N ratios in the released CO_2_ and N_2_ gases were determined using an ISOPRIME100 isotope ratio mass spectrometer (Isoprime, UK) and calibrated against IAEA and the United States Geological Survey (USGS) certified reference materials with known isotopic ratios: caffeine (IAEA‐600), graphite (USGS24) and potassium nitrate (USGS32). The δ^13^C_wood_ values (in ‰) were calculated as the deviation from the VPDB standard, while the δ^15^N_wood_ values were related to atmospheric nitrogen (AIR‐N_2_). Measurement accuracy, determined as the standard deviation (σ) of six replicate analyses on the same homogenized bulk wood sample, was better than 0.07‰ for δ^13^C and 0.11‰ for δ^15^N.

### 2.5 Climatic Data

The temperature data used in this study are based on the temperature reconstruction for Central Europe (Dobrovolný et al. 2010), which combines temperature indices based on documentary data from 1500 to 1854 ce and from instrumental measurements from 11 meteorological stations since 1760 ce. This series is fully representative of the territory of the Czech Republic (Brázdil et al. 2022a). Due to the high altitude of the Boubín reserve area (mean 1030 m asl), the reconstructed series were recalculated to the local position using a linear regression method, which was made possible by very high correlation coefficients for individual months since 1961 (Pearson r ranging from 0.93 to 0.98). From the monthly temperature series corrected to the Boubín position, mean annual and April–August temperatures for 1501–2020 ce (T_Apr‐Aug_) were then calculated and used in this study. To describe long‐term precipitation patterns, we used the standardized precipitation index for June–August (SPI_Jun‐Aug_) from Brázdil et al. (2016) This index was derived from reconstructed seasonal precipitation totals for the Czech Republic, combining precipitation indices from documentary data for 1501–1854 ce and Czech instrumental series from 1804 ce onwards (Dobrovolný et al. 2015).

### 2.6 Atmospheric Chemistry

To estimate historical nitrogen (N) deposition, we used a statistical approach that relied on the consistency between measured precipitation chemistry and the corresponding emission rates of NO_x_ and NH_3_ emissions in the Czech Republic since 1850. Spatial variations in precipitation chemistry were represented by empirically based interpolation, taking into account essential factors such as altitude, precipitation, and geographic coordinates (Oulehle et al. 2016; Treml et al. 2022). The estimate of N deposition before 1850 was made by comparing European and Czech N deposition between 1850 and 1930 and recalculated based on estimated European emissions since 1500 ce (Kopáček and Posch 2011). Reconstruction of precipitation pH since 1850 was made based on emission trends of dust, SO_2_, NO_x_, and NH_3_ as described in Kopáček et al. (2016). Precipitation pH before 1850 was arbitrarily chosen at 5.8, reflecting the weakly acidic nature of pre‐industrial rainwater. The corresponding concentrations of ambient CO_2_ were derived from Belmecheri and Lavergne (2020).

### 2.7 Calculation of Tree‐Ring iWUE and Δ^18^O_lw_

Intrinsic water‐use efficiency (iWUE) was calculated based on carbon isotope discrimination (Δ^13^C) estimated either for tree‐ring wood (iWUE_wood_) or cellulose (iWUE_cellulose_), depending on the analyzed material. Δ^13^C reflects carbon isotopic composition (δ^13^C) of a given sample along with the progressive depletion of ^13^C in atmospheric CO_2_ (δ^13^C_atm_) and post‐photosynthetic fractionation of organic material (2‰). Consequently, Δ^13^C values were used to calculate intercellular CO_2_ concentration (ci) taking into account also the effect of photorespiratory processes (Farquhar et al. 1982; Ubierna and Farquhar 2014) and individual fractionations associated with CO_2_ diffusion through stomata (4.4‰) (Craig 1953) and its carboxylation by the Rubisco enzyme (28‰) (Ubierna and Farquhar 2014). All calculations were performed using the *isocalcR* R package (Mathias and Hudiburg 2022), for an altitude of 1030 m asl and a leaf temperature corresponding to the T_Apr‐Aug_ (°C). The package incorporates recommended data for atmospheric CO_2_ concentration (c_a_) and δ^13^C_atm_ from 0 to 2021 ce, compiled by Belmecheri and Lavergne (2020), internally referencing this data within its functions. Therefore, the relatively constant δ^13^C_atm_ and CO_2_ before the end of the 19th century is implicitly considered in the iWUE calculations.

The oxygen isotope enrichment of leaf water above the source water (Δ^18^O_lw_) was estimated using the approach detailed in Guerrieri et al. (2019). This approach acknowledges the limitations of direct measurement of source water δ^18^O. As noted by Lin et al. (2022), the assumption that precipitation δ^18^O (δ^18^O_p_) directly reflects source water δ^18^O simplifies a complex hydrological system; source water δ^18^O can vary seasonally and among species due to differences in root systems and access to water sources. As a source of δ^18^O_p_ data, we used the site‐specific precipitation isotope time series prediction for the period 1950–2020 (Nelson et al. 2021) (https://isotope.bot.unibas.ch/PisoAI/). To extend this series to 1501 ce, we used the regression relationship between δ^18^O_p_ and site annual mean temperature, together with the annual SPI defined by the generalized linear model (GLM) output for the period 1950–2020 (*R* = 0.76, *p* < 0.001, *N* = 71). We then calculated the tree‐ring cellulose ^18^O enrichment above the source water (Δ^18^O_tr_) as follows:

ΔOtr18=δOcellulose−(δOp)18181+δOp181000

The final calculation of the Δ^18^O_lw_ is as follows:

ΔOlw18=ΔOtr18−εwc1−pxpex

Where *ε*_wc_ defines the temperature‐dependent fractionation between water and organic matter, and *p*_x_ is the proportion of stem water at the site of cellulose synthesis (≈1) of which a given fraction of oxygen atoms (*p*_ex_) is exchanged. We calculated the mean *p*_ex_ as a function of the mean vapour pressure deficit (VPD; in kPa) of the site as *p*_ex_ = 0.36 × VPD + 0.13 (Martínez‐Sancho et al. 2023).

### 2.8 Statistical Analysis

Although the oldest tree in our study is older than 500 years (the first spruce tree ring from 1437 ce), the analyses are done with the post‐1500 tree rings that cover available environmental data (Oulehle et al. 2025). In addition, we first identified temporal breakpoints in the species‐specific iWUE, δ^15^N_wood_, Δ^18^O_lw_ and BAI_ln_ chronologies using the *segmented* R package (Muggeo 2017). A maximum of two breakpoints were identified by sequential hypothesis testing on chronologies restricted to 1818–2020, so that the full number of tree replicates (35) were represented in the analysis. We then performed a full analysis of species‐specific annual iWUE_wood_, δ^15^N_wood_ and BAI (log‐transformed) chronologies (1501–2020) using a linear mixed effects (LME) model with environmental variables as fixed effects and tree ID as a random factor using the *nlme* package (Pinheiro et al. 2022). We allowed for interactions between CO_2_ and environmental data and between CO_2_ and cambial age (tree‐ring age) in the structure of the LME model. Each LME model allowed for temporal autocorrelation and was fitted by maximum likelihood. We selected the LME models based on the lowest corrected Akaike information criterion (ΔAICc < 2 compared to the best model) using the *MuMln* R package (Bartoń 2022) and averaged the model parameters across selected models to obtain the final conditional model. In addition, the best model (lowest ΔAICc) was fit by restricted maximum likelihood and used for visualization of the significant interaction effects between environmental factors on iWUE, δ^15^N_wood_ and BAI_ln_ using the R package *visreg* (Breheny and Burchett 2017). All continuous environmental variables used in the LME analysis were scaled to have a mean of zero and a standard deviation of one (Z‐score). To evaluate the degree of multicollinearity present in the LME models, we computed the variance inflation factor (VIF) for each continuous predictor using the car R package (Fox and Weisberg 2019). VIF values helped us assess the extent to which multicollinearity might impact the reliability of the model estimates, identifying any predictors exhibiting high levels of collinearity. We further assessed variation among climate, air chemistry, and tree ring variables using principal component analysis (PCA). We utilized the same continuous environmental variables included in the LME analysis. The PCA was conducted for each tree species separately, and the *factoextra* R package (Kassambara and Mundt 2020) was employed to summarize and visualize the multivariate data with principal components. All statistical analyses were performed in the RStudio 2023.12.1.402 (Posit Team 2024), and graphics were designed using the R package *ggplot2* (Wickham 2016).

## 3 Results

**FIGURE 2:**
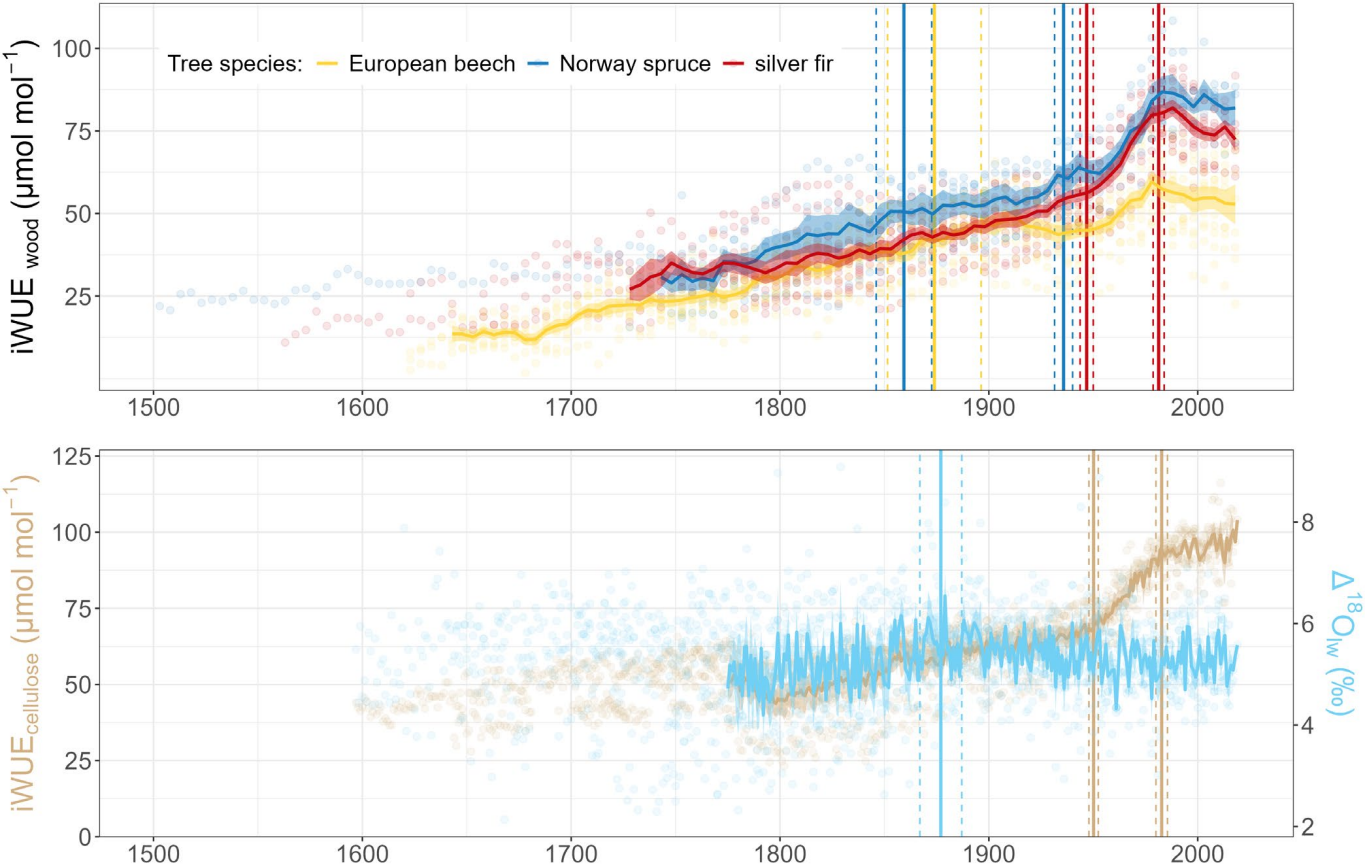
Individual species‐specific values (points) of intrinsic water‐use efficiency (iWUE_wood_) measured in aggregated 5‐year tree‐ring segments and intrinsic water‐use efficiency of Norway spruce measured in annual tree‐ring cellulose (iWUE_cellulose_) together with values of oxygen isotope leaf water enrichment above source water (Δ^18^O_lw_). Points are fitted with an average line starting in the year when the number of trees for each species reached at least 50% of all individuals analyzed (iWUE_wood_) and at 100% of all individuals analyzed (iWUE_cellulose_). Shading corresponds to the standard error of the mean. Vertical species‐specific lines show the significant change in trends over time (breakpoint analysis) and dashed lines show the standard error associated with the breakpoint estimate.

Over the last two centuries, intrinsic water‐use efficiency (iWUE_wood_) has increased significantly by 0.18 ± 0.01 μmol mol^−1^ year^−1^ (mean ± standard error; *p* < 0.001) across all species. Conifers showed the largest increases: Norway spruce at 0.22 ± 0.02 μmol mol^−1^ year^−1^ and silver fir at 0.21 ± 0.02 μmol mol^−1^ year^−1^, while European beech exhibited the lowest increase at 0.11 ± 0.01 μmol mol^−1^ year^−1^ (Figure 2). Since the early 19th century, iWUE_wood_ increased disproportionately—by 53% for beech, 89% for spruce, and 100% for fir. This increase was not monotonic and varied with ontogenetic development and atmospheric chemistry changes.

For beech, a significant breakpoint in iWUE_wood_ trend was identified around 1874 ± 22 years, with a pre‐1874 iWUE_wood_ rate of 0.16 ± 0.02 μmol mol^−1^ year^−1^ declining to 0.09 ± 0.011 μmol mol^−1^ year^−1^ post‐1874. Norway spruce iWUE_wood_ exhibited two key breakpoints in 1859 ± 13 years and 1936 ± 4 years, with initial iWUE_wood_ increasing at 0.22 ± 0.03 μmol mol^−1^ year^−1^, slowing to 0.09 ± 0.03 μmol mol^−1^ year^−1^ between 1860 and 1936, then sharply rising to 0.48 ± 0.03 μmol mol^−1^ year^−1^ past 1936. Silver fir iWUE_wood_ showed breakpoints in 1947 ± 3 years and 1981 ± 3 years, with initial iWUE_wood_ rising by 0.14 ± 0.01 μmol mol‐^1^ year^−1^, accelerating to 0.77 ± 0.09 μmol mol^−1^ year^−1^, and then declining by −0.22 ± 0.08 μmol mol^−1^ year^−1^ post‐1981. The intercellular to ambient CO_2_ concentration ratio (c_i_/c_a_) declined monotonically until the late 19th century, followed by varying perturbations in the 20th century (Figure S1). Complementary analysis of iWUE_cellulose_ in Norway spruce showed similar patterns, with breakpoints at 1950 ± 2 years and 1983 ± 3 years: pre‐1950 iWUE_cellulose_ increased at 0.13 ± 0.01 μmol mol^−1^ year^−1^, then accelerated to 0.75 ± 0.07 μmol mol^−1^ year^−1^, continuing at 0.13 ± 0.06 μmol mol^−1^ year^−1^ post‐1983. Δ^18^O_lw_ trends showed a breakpoint at 1877 ± 10 years, with an initial increase rate of 0.007‰ ± 0.002‰ year.^−1^ followed by a continuous decrease at −0.003‰ ± 0.0008‰ year^−1^ (Figure 2). The δ^18^O_cellulose_ and Δ^18^O_lw_ were highly correlated (*R* = 0.95, *p* < 0.001), with a δ^18^O_cellulose_ breakpoint at 1879 ± 14 years.

Environmental variable breakpoints were identified, with atmospheric CO_2_ concentration accelerating post‐1966 at 14.2 ± 0.2 ppm per decade, growing season air temperature (*T*_Apr‐Aug_) increasing post‐1974 at 0.44°C ± 0.09°C per decade, while no breakpoint emerged for standardized precipitation index (SPI_Jun‐Aug_). Precipitation acidity peaked around 1988, with a subsequent rise in pH at 0.4 ± 0.01 pH units per decade. Similarly, N deposition peaked around 1989, declining by −1.8 ± 0.1 kg N per decade (SI Figure 2). Additionally, VIF analysis indicated that N deposition and pH displayed significant multicollinearity (VIF > 5). This multicollinearity arises from the nature of atmospheric acidic deposition in Europe, peaking in the 1980s (Kopáček et al. 2016).

Linear mixed effects (LME) models for each tree species explained 79%–90% of iWUE variability (conditional *R* ^2^) (Table 1). Strong positive relationships with cambial (tree‐ring) age and N deposition were identified for all species. For conifers, iWUE positively relates to c_a_, whereas in beech, the positive N deposition effect on iWUE was attenuated at high c_a_ (SI Figure 3). In Norway spruce, the N deposition effect was strengthened with higher c_a_ (Figure S4A), and both conifers showed enhanced iWUE with low precipitation pH at increasing c_a_ (Figure S4B). Norway spruce also responded to SPI_Jun‐Aug_, accelerating iWUE with increasing c_a_ (Figure S4C), while the positive effect of cambial age on iWUE was reversed with increasing c_a_ (Figure S4D). iWUE_cellulose_ for Norway spruce tree rings was 9.6 ± 5.0 μmol mol^−1^ higher than iWUE_wood_ from bulk wood (*p* = 0.058; Table 1). The average iWUE_wood_ values were 37 ± 2.4 μmol mol^−1^ for European beech, 50 ± 3.3 μmol mol^−1^ for Norway spruce, and 47 ± 1.8 μmol mol^−1^ for silver fir over their lifetimes. Although the influence of climatic factors on iWUE was relatively limited, environmental factors combined explained 67%–78% of the total variance (marginal R^2^).

**FIGURE 3:**
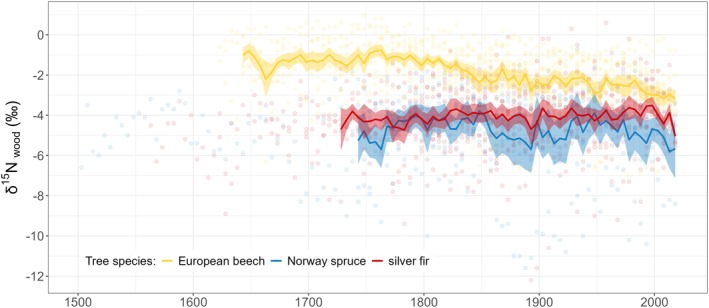
Individual species‐specific values (points) of δ^15^N_wood_ measured in aggregated 5‐year tree‐ring segments. Points are fitted with a mean line starting in the year when the number of trees for each species reached at least 50% of all analyzed individuals. Shading corresponds to the standard error of the mean.

For δ^15^N_wood_, European beech showed a significant decline by −0.006‰ ± 0.001‰ year^−1^ since 1818, while no significant trends were observed in conifers (Figure 3). LME models showed a strong negative relationship between cambial age and δ^15^N_wood_ across all species (Table 2). In silver fir, this relationship weakened with increasing c_a_. European beech exhibited a direct negative effect of N deposition on δ^15^N_wood_, which reversed with increasing c_a_ (Figure S5). Norway spruce showed a positive relationship between N deposition and δ^15^N_wood_, while in silver fir, the negative effect strengthened with increasing c_a_ (Figure S7A). A direct negative relationship between c_a_ and δ^15^N_wood_ was found for beech, and decreasing precipitation pH increased δ^15^N_wood_ in silver fir with increasing c_a_ (Figure S7B). SPI_Jun‐Aug_ positively relates to δ^15^N_wood_ in beech, interacting with c_a_ in spruce (SI Figure S6). For silver fir, the negative relationship of δ^15^N_wood_ to T_Apr‐Aug_ increased with rising c_a_ (Figure S7C), and low SPI_Jun‐Aug_ led to more negative δ^15^N_wood_ with increasing c_a_ (Figure S7D). Mean δ^15^N_wood_ values were − 2.0‰ ± 0.3‰ for beech, −4.5‰ ± 0.3‰ for spruce, and −4.2‰ ± 0.3‰ for fir. The total explained variability in δ^15^N_wood_ by LME models ranged from 50% to 57% (conditional *R* ^2^), with 14%–44% accounted for by environmental factors (marginal *R* ^2^) (Table 2).

**TABLE 2:** Parameter estimates (including interaction with air CO_2_) of the average linear mixed effects (LME) model identifying environmental factors influencing δ^15^N_wood_ of European beech, Norway spruce, and silver fir since 1501 CE. The LME model results shown are for the average conditional model, according to parameters for the final models of European beech (*N* = 12), Norway spruce (*N* = 16) and silver fir (*N* = 8) (models with ΔAIC_c_ < 2 derived from the best model with the lowest AIC_c_). *R* ^2^ values shown are for the best model; the marginal *R* ^2^ describes the variance in iWUE explained by fixed effects only, while the conditional *R* ^2^ represents the variance explained by fixed and random effects.

Predictors	European beech δ^15^N_wood_			Norway spruce δ^15^N_wood_			silver fir δ^15^N_wood_		
	Estimate ± SE	*z*‐value	*p*‐value	Estimate ± SE	*z*‐value	*p*‐value	Estimate ± SE	*z*‐value	*p*‐value
Intercept (‰)	−2.02 ± 0.30	6.74	< 0.001	−4.49 ± 0.31	14.3	< 0.001	−4.18 ± 0.28	14.8	< 0.001
Cambial age (year)	−0.53 ± 0.23	2.27	0.023	−1.26 ± 0.24	5.16	< 0.001	−0.66 ± 0.25	2.67	0.008
Air CO_2_ (ppm)	−0.36 ± 0.16	2.22	0.026	0.20 ± 0.26	0.79	0.432	0.47 ± 0.28	1.71	0.087
N deposition (kg ha^−1^ year^−1^)	−0.36 ± 0.13	2.73	0.006	0.63 ± 0.24	2.61	0.009	0.20 ± 0.25	0.80	0.423
pH precipitation	−0.05 ± 0.15	0.35	0.725	—	—	—	−0.02 ± 0.18	0.13	0.899
SPI_Jun‐Aug_	0.05 ± 0.03	2.00	0.045	−0.04 ± 0.05	0.81	0.417	0.00 ± 0.05	0.21	0.837
*T*_Apr‐Aug_ (°C)	−0.07 ± 0.04	1.62	0.106	0.09 ± 0.09	1.06	0.291	−0.11 ± 0.06	1.72	0.086
Air CO_2_: Cambial age	—	—	—	−0.17 ± 0.17	1.01	0.315	0.27 ± 0.08	3.37	< 0.001
Air CO_2_: N deposition	0.33 ± 0.10	3.36	< 0.001	−0.27 ± 0.21	1.29	0.196	−0.58 ± 0.22	2.62	0.009
Air CO_2_: pH precipitation	—	—	—	—	—	—	−0.50 ± 0.19	2.70	0.007
Air CO_2_: SPI_Jun‐Aug_	0.03 ± 0.03	0.77	0.440	0.15 ± 0.07	2.13	0.033	−0.13 ± 0.06	2.24	0.025
Air CO_2_: *T*_Apr‐Aug_	−0.03 ± 0.04	0.72	0.469	—	—	—	−0.16 ± 0.07	2.24	0.025
Observations	832			377			677		
Marginal *R* ^2^	0.19			0.44			0.14		
Conditional *R* ^2^	0.57			0.55			0.50		

Abbreviations: *p*‐value, two‐tailed; SE, standard error; *z*‐value, Wald chi‐squared test.

**FIGURE 4:**
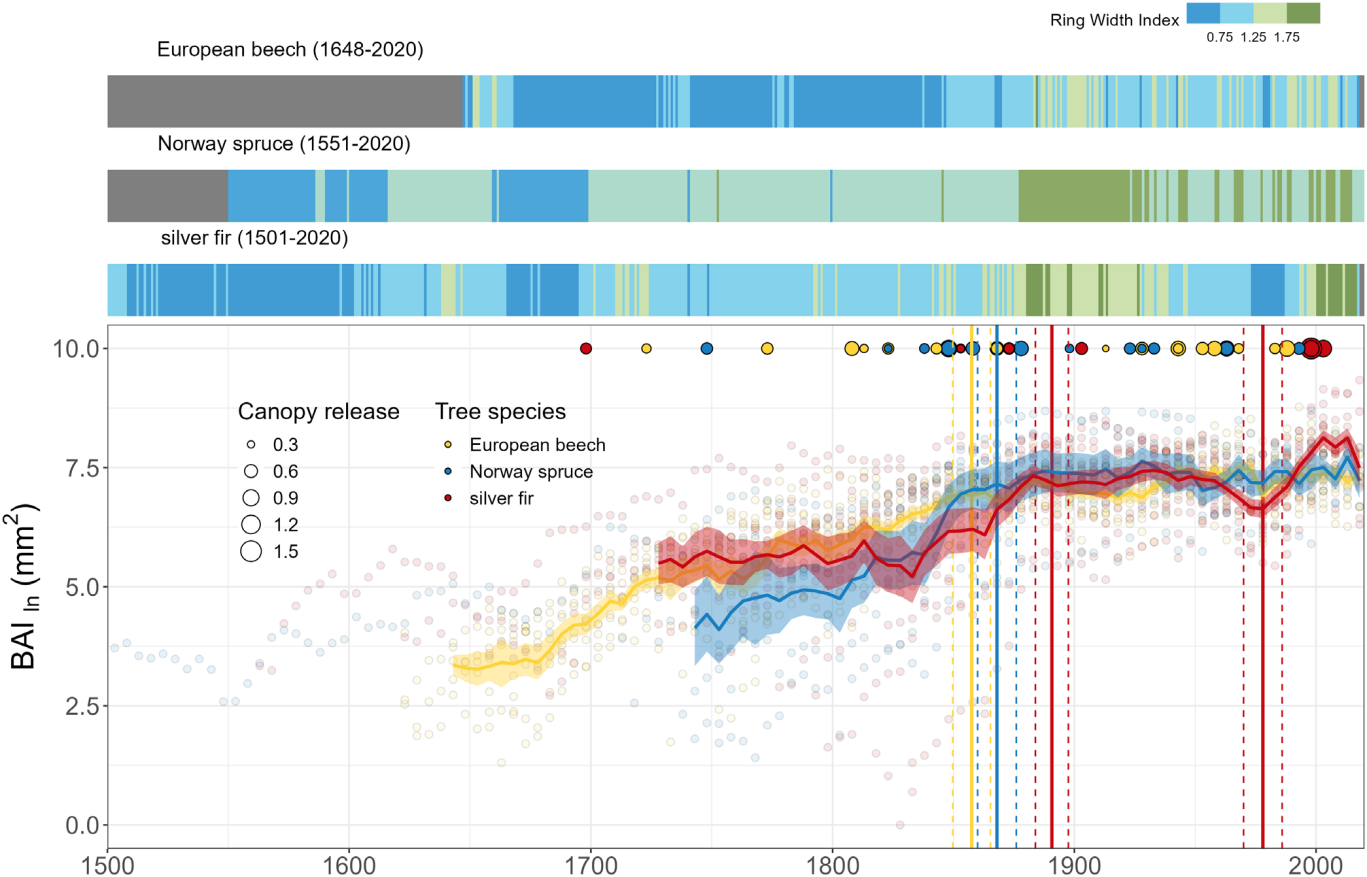
Species‐specific site chronologies for European beech, Norway spruce, and silver fir (top panels), adapted from Kašpar et al. (2021), with grey areas indicating no data. The bottom panel shows species‐specific transformed basal area increments (BAI_ln_) for individual trees used in isotope analyzes. Individual points are fitted with the mean line starting in the year when the number of trees for each species reached at least 50% of all analyzed individuals. The shading corresponds to the standard error of the mean and the vertical lines highlight temporal breakpoints when there was a significant change in the trend of BAI_ln_. The dashed vertical lines correspond to the uncertainty expressed by the standard error. Varying point sizes in the bottom panel indicate the growth release rate derived from the ring‐width data used in the chronologies above.

The de‐trended tree‐ring width index, from 92 beech trees, 1470 spruce trees, and 32 fir trees, indicated substantial growth acceleration post‐1870 (Figure 4). This growth persisted in beech and spruce through the 20th century, but fir growth reduced significantly in the latter half. Breakpoints in basal area increment (BAI_ln_) occurred at 1858 ± 8 for beech, with an initial increase rate of 0.016 ± 0.001 mm^2^ year^−1^ slowing to 0.003 ± 0.0007 mm^2^ year^−1^. For spruce, a breakpoint at 1868 ± 8 showed an initial rate of 0.02 ± 0.002 mm^2^ year^−1^ slowing to 0.002 ± 0.001 mm^2^ year^−1^. Fir showed breakpoints at 1891 ± 7 and 1978 ± 8 years, with initial increases at 0.03 ± 0.004 mm^2^ year^−1^, declining to −0.003 ± 0.003 mm^2^ year^−1^ until 1978, then increasing again at 0.03 ± 0.009 mm^2^ year^−1^. Stem growth release in fir mirrored spruce, primarily between 1840 and 1880 and around 2000 (Figure 4).

The LME models explained 54%–71% of the total variability in BAI_ln_ (marginal *R* ^2^) (Table 3). Cambial age was positively associated with stem increment across all species, with notable increases during periods of low atmospheric CO_2_ (as illustrated in Figures S8A–S10A). In beech, the BAI_ln_ was more responsive in younger tree rings, particularly as atmospheric CO_2_ levels increased. Nitrogen deposition positively affected BAI_ln_ in spruce, while c_a_ had a positive effect in fir. Both conifers showed strong positive relationships between BAI_ln_ and precipitation pH, further influenced by cambial age in fir (Figure S10B). For beech, BAI_ln_ was positively related to T_Apr‐Aug_, particularly during periods of high c_a_ (Figure S8B). For spruce, stem growth was positively related to SPI_Jun‐Aug_, and this relationship strengthened with c_a_ increases (Figure S9B). Silver fir showed no significant effects of climatic factors on BAI_ln_.

**TABLE 3:** Parameter estimates (including interaction with air CO_2_) of the average linear mixed effects (LME) model identifying environmental factors influencing basal area increment (BAI, natural log transformed) of European beech, Norway spruce, and silver fir since 1501 CE. The LME model results shown are for the average conditional model, according to parameters for the final models of European beech (*N* = 12), Norway spruce (*N* = 16) and silver fir (*N* = 8) (models with ΔAIC_c_ < 2 derived from the best model with the lowest AIC_c_). *R* ^2^ values shown are for the best model; the marginal *R* ^2^ describes the variance in iWUE explained by fixed effects only, while the conditional *R* ^2^ represents the variance explained by fixed and random effects.

Predictors	European beech BAI			Norway spruce BAI			silver fir BAI		
	Estimate ± SE	*z*‐value	*p*‐value	Estimate ± SE	*z*‐value	*p*‐value	Estimate ± S.E.	*z*‐value	*p*‐value
Intercept (ln, mm^2^)	6.43 ± 0.17	38.5	< 0.001	6.70 ± 0.30	22.1	< 0.001	6.76 ± 0.22	30.2	< 0.001
Cambial age (year)	1.26 ± 0.20	6.30	< 0.001	1.49 ± 0.21	7.21	< 0.001	1.47 ± 0.24	6.13	< 0.001
Air CO_2_ (ppm)	0.43 ± 0.23	1.85	0.064	0.26 ± 0.19	1.38	0.169	0.37 ± 0.16	2.32	0.020
N deposition (kg ha^−1^ year^−1^)	−0.17 ± 0.12	1.38	0.168	0.31 ± 0.15	2.03	0.042	−0.27 ± 0.16	1.68	0.094
pH precipitation	0.04 ± 0.10	0.42	0.676	0.45 ± 0.12	3.65	< 0.001	0.32 ± 0.11	2.81	0.005
SPI_Jun‐Aug_	—	—	—	0.04 ± 0.02	2.25	0.025	0.01 ± 0.001	1.14	0.255
*T*_Apr‐Aug_ (°C)	0.04 ± 0.02	2.39	0.017	0.04 ± 0.02	1.68	0.093	—	—	—
Air CO_2_: Cambial age	−0.57 ± 0.12	4.77	< 0.001	−0.46 ± 0.10	4.39	< 0.001	−0.46 ± 0.11	4.18	< 0.001
Air CO_2_: N deposition	−0.07 ± 0.10	0.71	0.476	−0.03 ± 0.10	0.27	0.786	−0.13 ± 0.12	1.14	0.255
Air CO_2_: pH precipitation	—	—	—	—	—	—	−0.20 ± 0.07	2.63	0.008
Air CO_2_: SPI_Jun‐Aug_	—	—	—	0.07 ± 0.02	3.28	0.001	—	—	—
Air CO_2_: T_Apr‐Aug_	0.05 ± 0.02	2.86	0.004	−0.01 ± 0.02	0.60	0.546	—	—	—
Observations	832			377			677		
Marginal *R* ^2^	0.71			0.55			0.54		
Conditional *R* ^2^	0.75			0.72			0.54		

Abbreviations: *p*‐value, two‐tailed; SE, standard error; *z*‐value, Wald chi‐squared test.

Principal component analysis (PCA) further revealed distinct patterns of variation in environmental variables and tree‐ring characteristics across European beech, Norway spruce, and silver fir (Figure S11). In European beech, PC1 was strongly associated with iWUE, N deposition, c_a_, and precipitation pH; PC2 was primarily driven by temperature variations (*T*_Apr‐Aug_); and PC3 highlighted relationships between SPI_Jun‐Aug_ and BAI_ln_. In silver fir, PC1 showed strong correlations between iWUE, N deposition, c_a_, and precipitation pH; PC2 was influenced by δ^15^N and SPI_Jun‐Aug_; and PC3 reflected the combined effects of SPI_Jun‐Aug_, *T*_Apr‐Aug_, δ^15^N, and BAI_ln_. In Norway spruce, PC1 was dominated by iWUE, N deposition, c_a_, and precipitation pH; PC2 was strongly associated with δ^15^N and cambial age; and PC3 reflected the influence of T_Apr‐Aug_ and BAI_ln_ (SI Table 2). These results demonstrate species‐specific associations among environmental drivers, shaping both physiological traits (iWUE, δ^15^N) and growth patterns (BAI_ln_).

## 4 Discussion

This study leverages tree‐ring carbon, oxygen, and nitrogen isotope signatures to assess the impacts of changing environmental conditions on intrinsic water‐use efficiency (iWUE) and nitrogen (N) dynamics over the past 500 years in three dominant tree species of a temperate mountain primeval forest. The unique long‐term dataset reveals that atmospheric chemistry and climate dynamics are nonlinear and have shifted fundamentally since the latter half of the 20th century. Significant disturbance events around post‐1840 marked the first major break in the data, coinciding with the end of the Little Ice Age. These disturbances, occurring within the cyclical dynamics of mountain temperate forests, peaked in the mid‐19th century alongside rising temperatures and wetter conditions. While these disturbances align with natural development cycles of the forest (Kašpar et al. 2020), the changing climate—particularly alterations in rainfall and temperature—could have played a role in shaping these events. During this period, trees emerged from shading and reached the main canopy (Brázdil et al. 2022b), modulating carbon and water fluxes and altering growth dynamics. The Industrial Revolution accelerated changes in atmospheric and climatic conditions, resulting in varying degrees of physiological acclimation among tree species in response to the new environmental conditions. This shift is reflected in altered species representation and growth dynamics, with European beech showing increased dominance over Norway spruce, and silver fir exhibiting improved growth after decades of suppressed vitality caused by acid air pollution. While some studies report drought‐induced fir decline (Linares and Camarero 2012), our findings align with broader patterns of forest assemblage shifts (Peñuelas et al. 2007; Elling et al. 2009; Bolte et al. 2010; Cavlovic et al. 2015; Kulla et al. 2023).

Old trees selected for this study, inherently associated with long‐term growth suppression, had initial iWUE_wood_ values below 30 μmol mol^−1^ during the 16th and 17th centuries. This indicates a positive effect of cambial age on iWUE_wood_, particularly before trees reached the main canopy and before significant atmospheric pollution, including increased CO_2_ concentration, N deposition, and precipitation acidity. The increase in iWUE_wood_ during developmental growth, despite stable CO_2_ levels, suggests a decline in c_i_ to c_a_ ratio. This likely reflects adjustments in stomatal and mesophyll conductance, potentially linked to leaf morphological changes in response to varying light intensities within the canopy (Cano et al. 2013). The slowed iWUE increase during the 1840–1880 disturbance period, concurrent with accelerated BAI, highlights interactions between developmental growth stages and iWUE. This aligns with observed correlations between tree height, altered crown illumination (possibly due to increased light penetration after disturbance), and iWUE, highlighting the influence of light and water stress on photosynthetic acclimation (Bert et al. 1997; McDowell et al. 2011; Brienen et al. 2017; Kašpar et al. 2024).

For Norway spruce, changes in iWUE and BAI trends align with changes in stomatal conductance (g_s_), additionally inferred from Δ^18^O_lw_ (Guerrieri et al. 2019; Mathias and Thomas 2021). The dual‐isotope concept suggests that iWUE increases during suppressed growth were due to reduced g_s_, or a reduction in g_s_ combined with A_net_ gain (Siegwolf et al. 2023). The prolonged shading conditions led to the development of leaves with typically lower δ^13^C compared to sunlit leaves, a phenomenon observed across all three studied species (Schleser and Jayasekera 1985; Koch et al. 2004; Klesse et al. 2018). The developmental effect on tree‐ring δ^13^C_wood_ was significant, diminishing as trees transitioned from suppression to main canopy‐stage growth. This transition period was marked by heavy windstorms (Brázdil et al. 2017, 2018) frequently followed by significant bark beetle disturbances post‐1840 (Brázdil et al. 2022b).

Across all species, increasing tree age was inversely related to δ^15^N_wood_, likely reflecting greater reliance on ectomycorrhizal (EM) N uptake and potential changes in soil microbiome composition (Clemmensen et al. 2015; Wasyliw and Karst 2020; Birch et al. 2021). However, our experimental design does not allow us to fully disentangle these factors. The substantial variation in microbiome structure necessitates caution when interpreting δ^15^N changes solely as a function of EM activity. Nevertheless, EM fungi efficiently fractionate N isotopes, supplying ^15^N‐depleted N to the host tree (Hobbie and Ouimette 2009). Before industrial pollution, European beech exhibited higher δ^15^N_wood_ than conifers (slightly lower signature than atmospheric N_2_), indicating N uptake from deeper soil horizons with relative ^15^N enrichment or primarily from N fixation under low N deposition. Upon reaching canopy cover, environmental changes tied to atmospheric pollution became more prominent, revealing significant interspecific differences in leaf gas exchange and δ^15^N_wood_ characteristics.

The success of European beech in adapting to changing environmental conditions lies in its ability to exploit eutrophic soil conditions effectively. The relationship between N deposition and iWUE_wood_ was notably CO_2_ concentration‐dependent. At lower atmospheric CO_2_ concentrations, increasing N deposition tended to have a positive effect on iWUE_wood_, marked by a decrease in δ^15^N_wood_. As CO_2_ levels rose, the effect of N deposition on δ^15^N_wood_ reversed, leading to higher δ^15^N_wood_ values—a phenomenon likely related to progressive N saturation and increased ^14^N losses from the ecosystem (Houlton and Bai 2009; Fang et al. 2015; Oulehle et al. 2021). Wetter climate conditions further amplified this effect.

The proposed presence of arbuscular mycorrhiza (AM) in European beech (Hodge and Storer 2014) permitted efficient N uptake from deposition, facilitating growth during the early industrial period. Notably, dual AM‐EM associations (Teste et al. 2020; Heklau et al. 2021) could enable beech to capitalize on the increased availability of inorganic N and maintain robust growth despite declines in N deposition. The gradual convergence of δ^15^N_wood_ values with conifers may further indicate a progressive deepening of the dependence of N uptake on the EM over AM symbiosis (Craine et al. 2009).

The coupling of stem growth and iWUE_wood_ in beech during rising air pollution suggests that N deposition stimulated net photosynthesis (Adams et al. 2021), supporting growth under favorable climatic conditions. This response is likely mediated by changes in leaf‐level nitrogen and carbon isotope discrimination, as observed in another study (Aranda et al. 2017). Despite recent declines in N deposition, beech stem growth has remained stable, suggesting the species´ capacity to leverage eutrophic soils and a warmer climate.

In stark contrast, Norway spruce exhibited high values of iWUE, especially under increased atmospheric acidity, as observed elsewhere (Savard et al. 2004; Mathias and Thomas 2018). While increased N deposition and atmospheric CO_2_ positively influenced iWUE_wood_, precipitation pH had a negative effect. This negative effect likely stems from pollutant‐induced, particularly SO_2_‐induced, stomatal closure and reduced photosynthesis rates (Savard et al. 2004). The increased iWUE likely reflects a greater reduction in g_s_ than in *A*_net_ (Boettger et al. 2014). Consequently, this negative impact on stomatal function, primarily driven by SO_2_, negatively affected growth.

Improving air quality over recent decades leveled iWUE_wood_, yet persistent iWUE saturation left Norway spruce vulnerable to drier conditions and exacerbated growth declines, making the species increasingly susceptible to climate change and biotic disturbances. This vulnerability was further highlighted by the strong correlation between BAI trends and moist conditions, suggesting spruce is well‐suited to disturbance‐prone environments where soil moisture and N availability increase temporarily (Oulehle et al. 2018; Kopáček et al. 2023). However, the constraining effect of iWUE saturation in drier climates diminished this advantage, leaving Norway spruce disadvantaged compared to deep‐rooted species like European beech and silver fir, which exhibited more flexible physiological responses to changing conditions.

Silver fir exhibited a distinct physiological response trajectory, paralleling Norway spruce's iWUE patterns but with notable differences in recent decades. The iWUE_wood_ of silver fir accelerated during the 20th century due to increased N deposition and atmospheric acidity. However, a noticeable decline in iWUE_wood_ over the past four decades contrasted with Norway spruce trends.

This trend reversal in fir, indicated by high contemporary stem growth rates, highlights the species' sensitivity to acidic pollution and its subsequent adaptation under cleaner air conditions. This contrasts sharply with reports of drought‐induced decline at the dry edge of the silver fir distribution in Europe (Linares and Camarero 2012), demonstrating the complex interplay of environmental stressors on fir growth. Nevertheless, the recent improvement aligns with broader patterns of forest assemblage shifts and strong contemporary regeneration of fir across Europe (Elling et al. 2009; Boettger et al. 2014; Büntgen et al. 2014). As pollution abated, silver fir no longer faced growth limitations, and its flexible adaptation of iWUE allowed for accelerated stem growth without apparent climatic constraints (Kašpar et al. 2021). Similar to European beech, silver fir benefits from increased N and CO_2_ availability, underscoring its potential for increased representation in future forest assemblages (Gbur et al. 2025).

This study adds to our understanding of how the interplay of environmental changes over 500 years have influenced tree physiology and growth in a temperate forest ecosystem. By integrating isotope signatures with growth dynamics, we provide a consistent framework for understanding the complex interactions between tree species competition and environmental changes. Our findings align with extensive previous research and reveal how coupled CO_2_ and N fertilization, conditioned by climatic constraints, affect tree functioning and forest ecosystem evolution (Thomas et al. 2013; Adams et al. 2021; Gharun et al. 2021; Mathias and Thomas 2021; Mathias et al. 2023).

Understanding species‐specific responses to environmental changes is crucial for forest management and conservation strategies. Our study highlights the resilience of European beech (Šebková et al. 2011) and the potential of silver fir under improved air quality conditions. In contrast, the vulnerability of Norway spruce to climate change and environmental disturbances emphasizes the need for diversified forest compositions to enhance ecosystem resilience. The strong developmental influences on growth, iWUE, and δ^15^N necessitate careful consideration when interpreting long‐term trends and conducting for example, dendroclimatological analyses. The study applied a holistic approach that integrates multiple environmental factors and provides an improved understanding of how atmospheric chemistry and climate changes affect tree functioning. These insights are vital for predicting the long‐term evolution of forest ecosystems and informing adaptive management strategies to sustain forest health and productivity in the face of ongoing environmental changes. Future research should expand isotopic and physiological studies across diverse forests to comprehensively capture global forest responses to environmental changes (Peñuelas et al. 2011; Walker et al. 2021). By combining historical and contemporary data, we can better anticipate and mitigate climate change impacts on forest ecosystems, ensuring their sustainability for future generations.

## Author Contributions

**Filip Oulehle:** conceptualization, funding acquisition, investigation, methodology, writing – original draft. **Pavel Šamonil:** conceptualization, investigation, methodology, writing – original draft. **Otmar Urban:** investigation, methodology, validation. **Josef Čáslavský:** investigation, methodology. **Alexander Ač:** methodology. **Ivana Vašíčková:** investigation. **Jakub Kašpar:** investigation. **Pavel Hubený:** investigation. **Rudolf Brázdil:** investigation, methodology. **Miroslav Trnka:** methodology, validation.

## Conflicts of Interest

The authors declare no conflicts of interest.

## Supporting information


Data S1.

## Data Availability

The data that support the findings of this study are openly available in figshare at https://doi.org/10.6084/m9.figshare.28589138. Temperature reconstruction since AD 1501 was obtained from the University of Bern Open Repository and Information System (BORIS) repository at https://doi.org/10.48620/167. Drought index (SPI) reconstruction since AD 1501 was obtained from figshare at https://doi.org/10.6084/m9.figshare.28601123.v1.
