# Supplementary material for: Growth and Assemblage Dynamics of Temperate Forest Tree Species Match Physiological Resilience to Changes in Atmospheric Chemistry
Source: Glob Chang Biol. 2025 Mar 26;31(3):e70147. doi: 10.1111/gcb.70147 (PMC11938019; doi:10.1111/gcb.70147)
Supplement: Supplementary file 1 — Data S1. [file GCB-31-e70147-s001.docx]

Supplementary Materials for

Growth and assemblage dynamics of temperate forest tree species match physiological resilience to changes in atmospheric chemistry

**Running head: Forest dynamics amidst shifting air chemistry**

Filip Oulehle^1,2,*^, Pavel Šamonil^3,4^, Otmar Urban^2^, Josef Čáslavský^2^, Alexander Ač^2^, Ivana Vašíčková^3^, Jakub Kašpar^3^, Pavel Hubený^5^, Rudolf Brázdil^2,6^, Miroslav Trnka^2^

*Corresponding author. Email: [filip.oulehle@geology.](mailto:filip.oulehle@geology.)cz

**Table S1.** Species-specific statistics for tree metrics. The metrics include Gl (Gleichläufigkeit), which measures the year-to-year synchronicity in growth changes between tree-ring series as a percentage. It also includes THO, derived from Student's t-test according to Hollstein, indicating the significance of the correlation coefficient while accounting for the length of overlap in tree-ring series. Age refers to the number of measured tree rings, and DBH represents the trunk diameter at breast height.

| **Species** | **Variable** | **Mean** | **Min** | **Max** |
| --- | --- | --- | --- | --- |
| European beech (N = 12) | THO | 5.56 | 1.47 | 8.61 |
|  | Gl (%) | 60.5 | 54.0 | 68.1 |
|  | Age (yrs) | 367 | 260 | 398 |
|  | DBH (mm) | 676 | 523 | 810 |
|  |  |  |  |  |
| Norway spruce (N=11) | THO | 8.15 | 3.56 | 13.3 |
|  | Gl (%) | 66.5 | 61.7 | 72.4 |
|  | Age (yrs) | 360 | 253 | 567 |
|  | DBH (mm) | 754 | 432 | 1115 |
|  |  |  |  |  |
| silver fir (N=12) | THO | 6.50 | 1.44 | 9.00 |
|  | Gl (%) | 64.6 | 53.5 | 68.1 |
|  | Age (yrs) | 308 | 203 | 451 |
|  | DBH (mm) | 766 | 450 | 1132 |

**Table S2**. **PCA contributions of environmental variables and tree ring characteristics across tree species.** Table presents the contributions of cambial age, standardized precipitation index (SPI_Jun-Aug_), growing season temperature (T_Apr-Aug_), nitrogen deposition, pH of precipitation, and atmospheric CO_2_ concentration alongside tree ring characteristics including δ^15^N, logarithm of basal area increment (BAI_ln_), and intrinsic water-use efficiency (iWUE) to the first three principal components derived from the PC analysis.

|  |  | European beech | | | Norway spruce | | | | silver fir | | | |
| --- | --- | --- | --- | --- | --- | --- | --- | --- | --- | --- | --- | --- |
| *Variable* | **PC** | **1** | **2** | **3** | | **1** | **2** | **3** | | **1** | **2** | **3** |
| *Cambial age* |  | 17.25 | 0.29 | 0.03 | | 7.53 | 34.42 | 0.53 | | 14.23 | 5.82 | 8.54 |
| *Air CO2* |  | 14.76 | 5.27 | 7.72 | | 18.06 | 0.45 | 3.34 | | 16.79 | 0.00 | 4.82 |
| *N deposition* |  | 17.91 | 1.07 | 0.44 | | 20.05 | 0.29 | 1.77 | | 17.22 | 4.65 | 0.01 |
| *pH precipitation* |  | 15.83 | 0.21 | 1.11 | | 19.23 | 0.13 | 0.64 | | 17.06 | 4.42 | 0.01 |
| *SPI _Jun-Aug_* |  | 0.00 | 36.14 | 48.40 | | 0.12 | 2.28 | 38.35 | | 0.10 | 25.62 | 25.68 |
| *T _Apr-Aug_* |  | 2.22 | 52.02 | 10.89 | | 3.37 | 1.85 | 46.43 | | 4.88 | 11.03 | 30.15 |
| *iWUE* |  | 14.73 | 1.66 | 7.31 | | 19.55 | 2.01 | 0.28 | | 18.85 | 0.20 | 0.03 |
| *δ^15^N* |  | 4.66 | 0.12 | 9.93 | | 0.21 | 56.89 | 1.91 | | 0.01 | 46.68 | 23.74 |
| *BAI_ln_* |  | 12.64 | 3.21 | 14.15 | | 11.88 | 1.68 | 6.74 | | 10.86 | 1.58 | 7.02 |


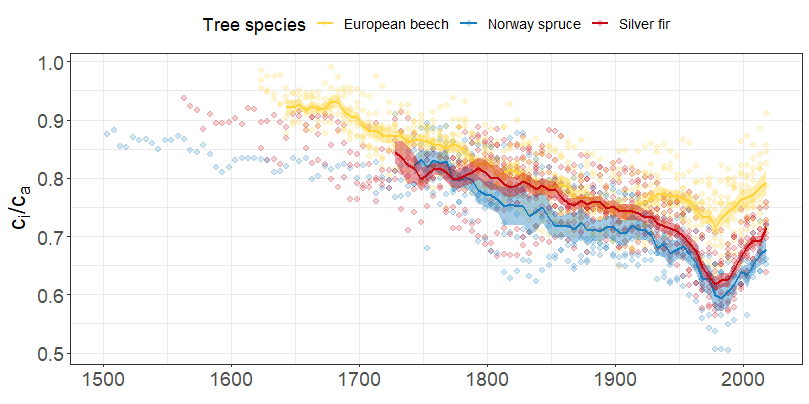


Fig. S1. Individual species-specific values (points) of the ratio of intercellular to ambient CO_2_ concentration (c_i_/c_a_) calculated for aggregated 5-year tree-ring segments. Points are fitted with a mean line starting in the year when the number of trees for each species reached at least 50% of all individuals analyzed. Shading corresponds to the standard error of the mean.


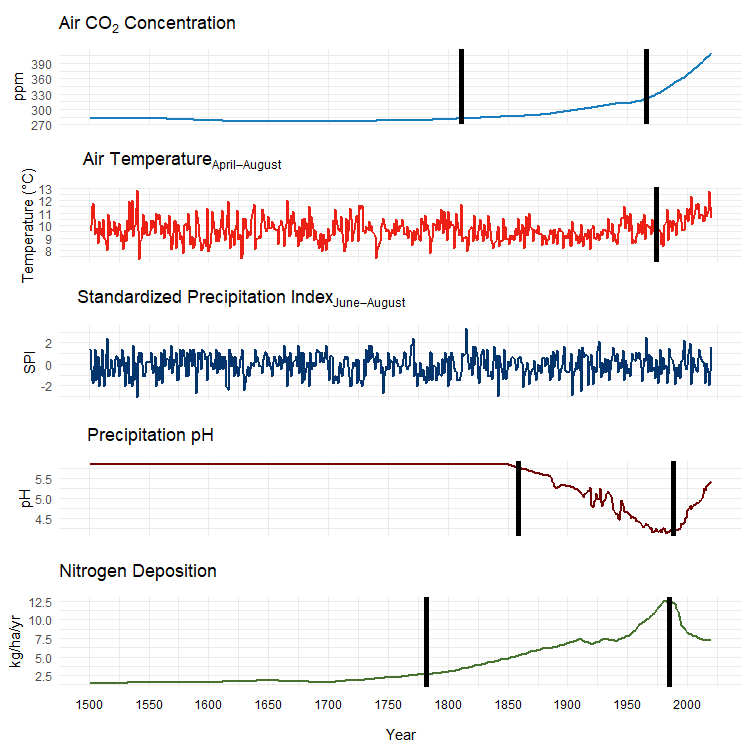


Fig. S2. Temporal patterns (1501-2020 CE) of environmental parameters with an indication of the years (vertical black line) when there was a significant change in the trend (identified by breakpoint analysis).


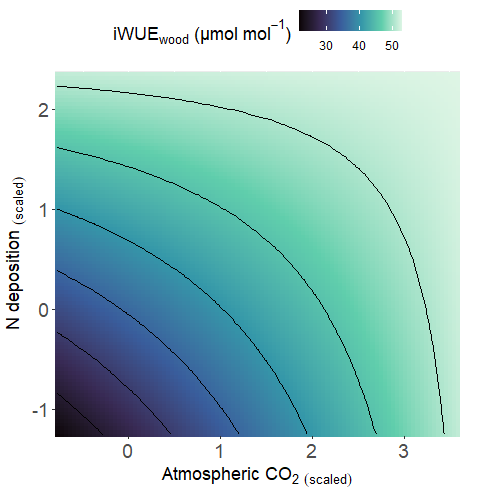


Fig. S3. Nature of the interaction between nitrogen (N) deposition and atmospheric CO_2_ on the intrinsic water-use efficiency (iWUE) of European beech trees. Values for each environmental factor were scaled by dividing the centred original values by their standard deviations. The parameter estimates for the interaction are presented in Table 1.


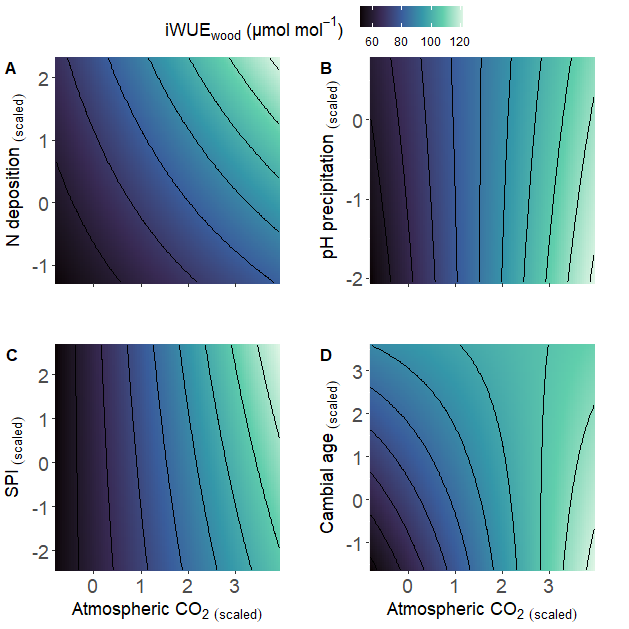


Fig. S4. Nature of the interactions between nitrogen (N) deposition and atmospheric CO_2_ (A), precipitation pH and atmospheric CO_2_ (B), June-August standardised precipitation index (SPI_Jun-Aug_) and atmospheric CO_2_ (C) and cambial age and atmospheric CO_2_ (D) on intrinsic water-use efficiency (iWUE) in Norway spruce trees. Values for each environmental factor were scaled by dividing the centred original values by their standard deviations. The parameter estimates for the interaction are presented in Table 1.


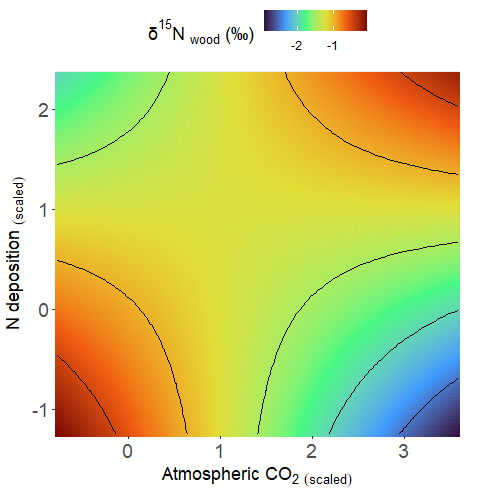


Fig. S5. Nature of the interaction between nitrogen (N) deposition and atmospheric CO_2_ on δ^15^N_wood_ in European beech trees. Values for each environmental factor were scaled by dividing the centred original values by their standard deviations. Parameter estimates for the interaction are presented in Table 2.


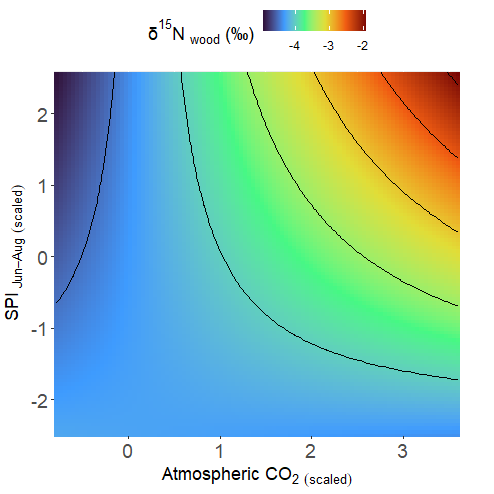


Fig. S6. Nature of the interaction between June-August standardised precipitation index (SPI_Jun-Aug_) and atmospheric CO_2_ on δ^15^N_wood_ in Norway spruce trees. Values for each environmental factor were scaled by dividing the centred original values by their standard deviations. Parameter estimates for the interaction are presented in Table 2.


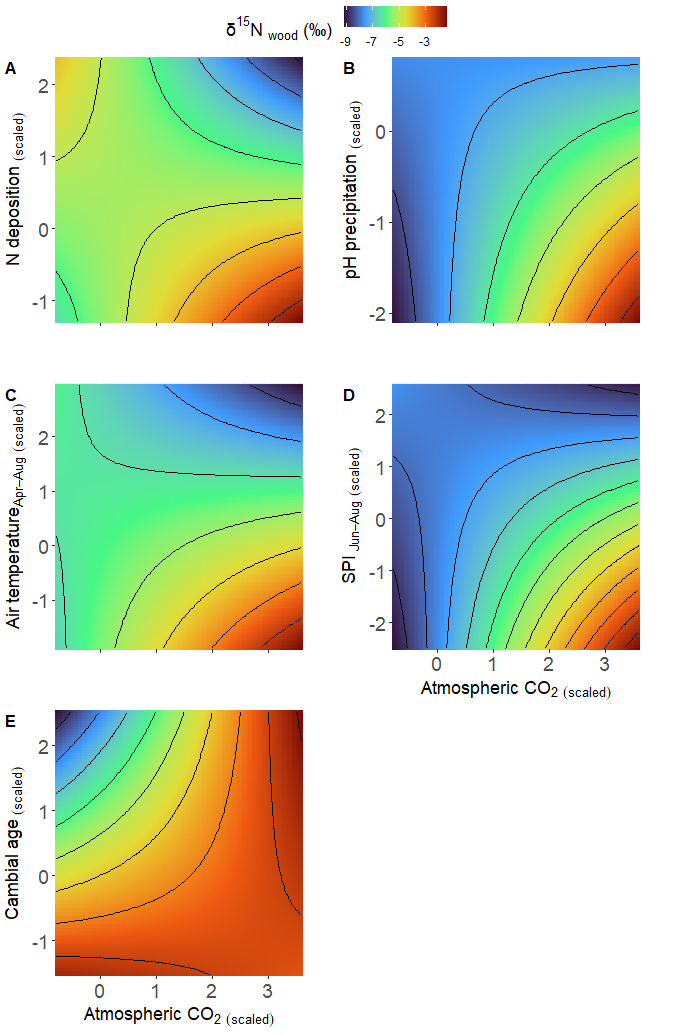


**Fig. S7.** Nature of the interactions between nitrogen (N) deposition and atmospheric CO_2_ (A), precipitation pH and atmospheric CO_2_ (B), April-August air temperature and atmospheric CO_2_ (C), June-August standardised precipitation index (SPI_Jun-Aug_) and atmospheric CO_2_ (D), and cambial age and atmospheric CO_2_ (E) on δ^15^N_wood_ in Silver fir. Values for each environmental factor were scaled by dividing the centred original values by their standard deviations. Parameter estimates for the interaction are presented in Table 2.


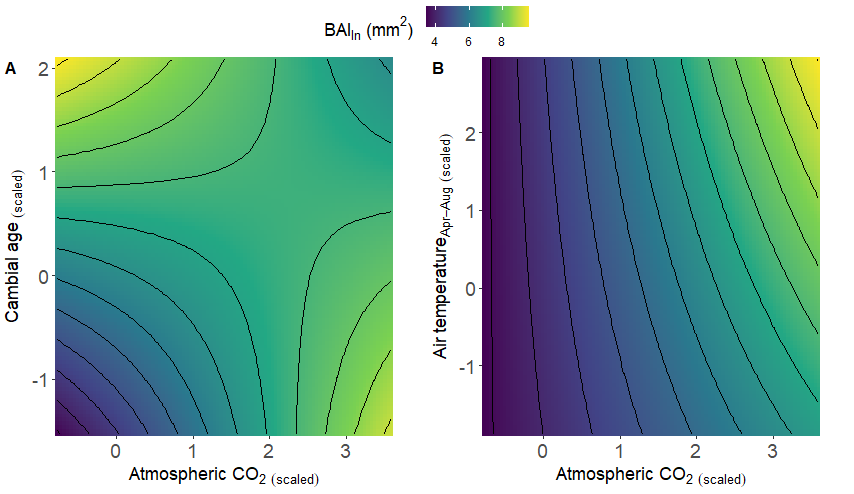


**Fig. S8.** Nature of the interactions between cambial age and atmospheric CO_2_ (A), April-August air temperature and atmospheric CO_2_ (B) on basal area increment (BAI) in European beech trees. The values for each environmental factor were scaled by dividing the centred original values by their standard deviations. Parameter estimates for the interaction are presented in Table 3.


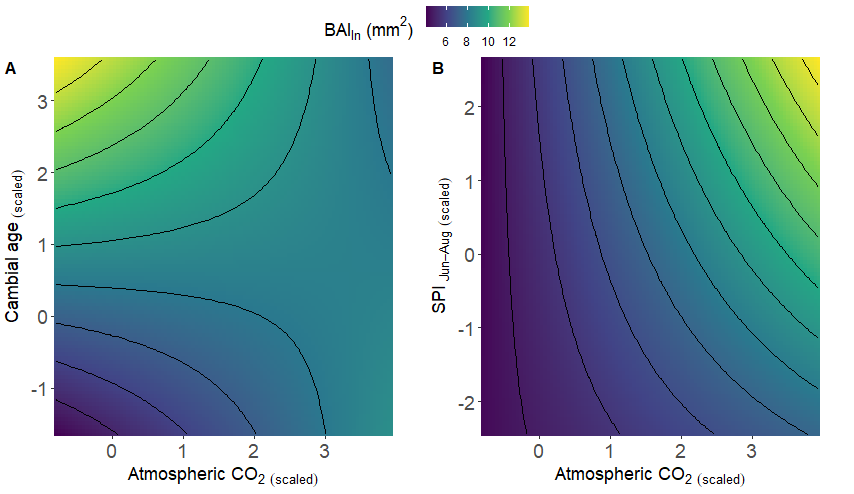


**Fig. S9.** Nature of the interactions between cambial age and atmospheric CO_2_ (A), June-August standardised precipitation index (SPI_Jun-Aug_) and atmospheric CO_2_ (B) on basal area increment (BAI) in Norway spruce trees. The values for each environmental factor were scaled by dividing the centred original values by their standard deviations. The parameter estimates for the interaction are presented in Table 3.


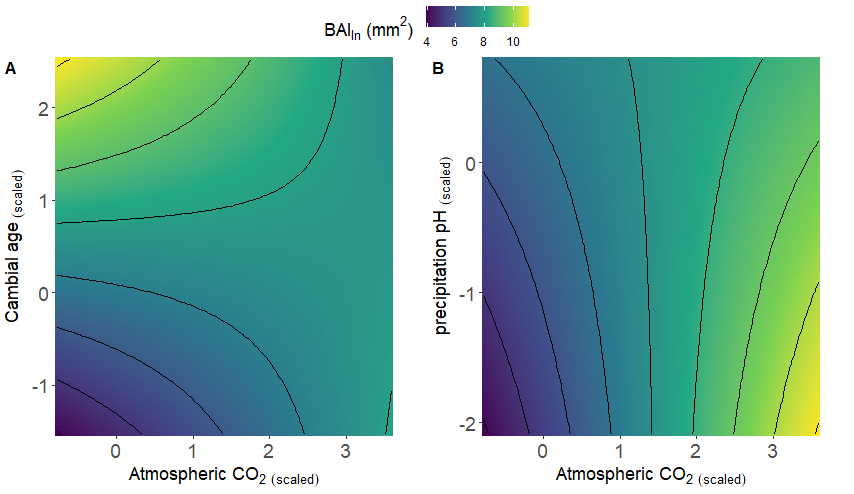


**Fig. S10.** Nature of the interactions between cambial age and atmospheric CO_2_ (A), precipitation pH and atmospheric CO_2_ (B) on basal area increment (BAI) in silver fir trees. The values for each environmental factor were scaled by dividing the centred original values by their standard deviations. The parameter estimates for the interaction are presented in Table 3.


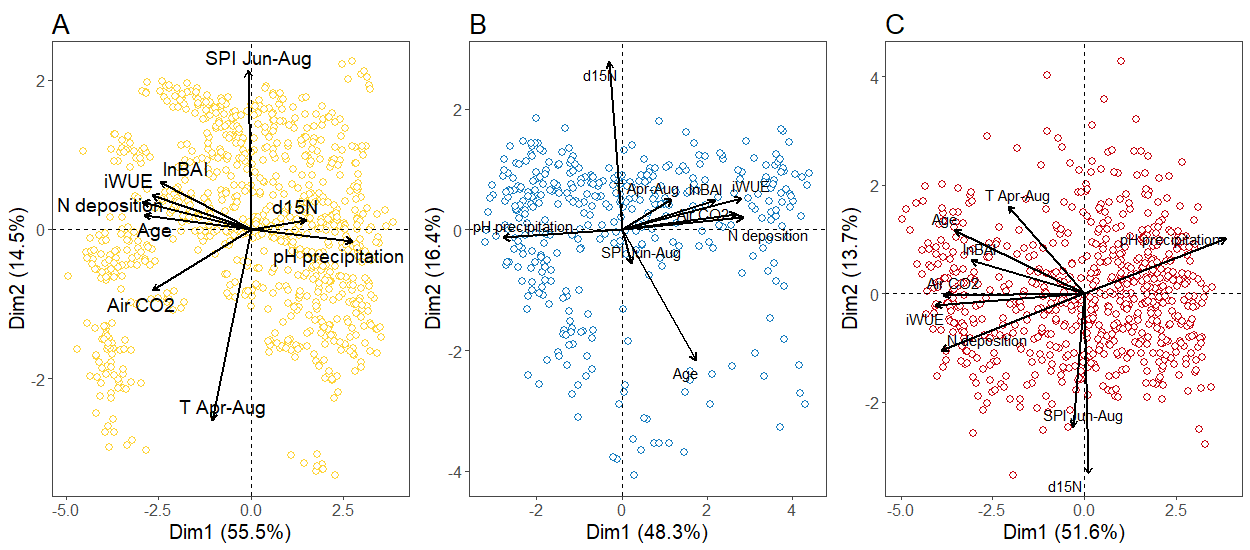


**Fig. S11**. Biplots illustrating the results of a principal component analysis (PCA) of environmental variables and tree ring characteristics for three tree species: European beech (A), Norway spruce (B), and silver fir (C). Each panel displays the scores of individual tree-rings plotted against the loadings of the variables. Dimension 1 (Dim1) and Dimension 2 (Dim2) represent the principal components (PC), with the percentage of variance explained shown in parentheses. Vector direction and length indicate the contribution and correlation of each variable to the principal components.
